# Extensive, Multifocal Pott Disease in a United States–Born Veteran Without Human Immunodeficiency Virus

**DOI:** 10.1093/ofid/ofaf398

**Published:** 2025-07-01

**Authors:** David D Arteaga, Reuben J Arasaratnam, Lisa Y Armitige, Donald F Storey

**Affiliations:** Department of Internal Medicine, University of Texas Southwestern Medical Center, Dallas, Texas, USA; Department of Internal Medicine, University of Texas Southwestern Medical Center, Dallas, Texas, USA; Veterans Affairs North Texas Health Care System, University of Texas Southwestern Medical Center, Dallas, Texas, USA; Heartland National Tuberculosis Center, University of Texas Health Center at Tyler, Tyler, Texas, USA; Department of Internal Medicine, University of Texas Southwestern Medical Center, Dallas, Texas, USA; Veterans Affairs North Texas Health Care System, University of Texas Southwestern Medical Center, Dallas, Texas, USA

**Keywords:** Pott disease, tuberculosis-associated immune reconstitution inflammatory syndrome, tuberculous involvement of the central nervous system, tuberculous spondylitis

## Abstract

*Mycobacterium tuberculosis* can infect the native vertebral body and nearby structures to cause tuberculous spondylodiscitis, also known as Pott disease. We present a case of extensive, multifocal Pott disease in a United States–born veteran without human immunodeficiency virus. We briefly comment on when to consider Pott disease in cases of vertebral osteomyelitis. We also discuss treatment considerations for Pott disease with and without tuberculous involvement of the central nervous system, the recognition of tuberculosis-associated immune reconstitution inflammatory syndrome, and certain indications for surgery in Pott disease.

Pott disease is infrequent in the United States (US). Clinicians should consider the disease in patients with vertebral osteomyelitis plus tuberculous risk factors or an initially nondiagnostic tissue biopsy [1]. The medical management of Pott disease with antitubercular therapy (ATT) is based on studies of pulmonary tuberculosis (PTB) and tuberculous meningitis (TBM) [2, 3]. Patients beginning ATT may experience a paradoxical worsening because of an immune phenomenon called tuberculosis-associated immune reconstitution inflammatory syndrome (TB-IRIS) [4]. In persons with human immunodeficiency virus (HIV), the incidence and morbidity of TB-IRIS have been mitigated by using adjunctive corticosteroids [5, 6]. Indications for surgery in Pott disease include spinal instability, neurologic compromise, and failed medical management [7]. Following the CAse REport (CARE) guidelines, we report an unusual case of Pott disease involving noncontiguous segments of the lumbar and thoracic spine in a US-born person without HIV and its successful treatment using central nervous system (CNS)–penetrant ATT and adjunctive corticosteroids [8].

## CASE PRESENTATION

A 61-year-old Veteran presented to an outside hospital with 1 year of low back pain, 40 pounds of weight loss, and 4 months of night sweats and lower extremity paresthesias. His medical history included allergic rhinitis and gastrointestinal reflux disease, and his medications were fluticasone, famotidine, and naproxen as needed. On arrival, he was afebrile and hemodynamically stable. Physical examination revealed tenderness of the lower back without evidence of myelopathy. Laboratory data showed a leukocyte count of 7.3 × 10^3^ cells/μL, erythrocyte sedimentation rate of 87 mm/hour, and C-reactive protein (CRP) of 13 mg/L. Contrast magnetic resonance imaging (MRI) of the lumbar spine noted enhancement of the L1 and L2 vertebral bodies, associated vertebral burst fractures, prevertebral abscesses extending to bilateral psoas muscles, and an epidural abscess causing central stenosis. Vancomycin, cefepime, and metronidazole were started for suspected pyogenic vertebral osteomyelitis. Neurosurgery was consulted, and the following interventions were done: laminectomy of L1 and L2, evacuation of the epidural abscess, and posterior fusion of T11 to L4 using bilateral pedicle screws. The abscess specimen was submitted for cytology, bacterial culture with Gram stain, fungal culture with smear, and acid-fast bacillus (AFB) culture with smear. Gram stain showed moderate white blood cells (WBCs) with no organisms. Smears were negative for fungal elements and acid-fast bacteria. Cytology returned negative, and intraoperative cultures detected no growth by 7 days.

The patient was therefore transferred to our facility to continue care and complete 6 weeks of empiric antibiotic therapy. Additional social history was notable for prior military service in Japan and occupation at a dairy processing plant for 2 decades. Given his epidemiologic risk factors, and since intraoperative cultures from the outside facility were negative, additional serum studies were collected to assess for less common causes of vertebral osteomyelitis: *Brucella* antibody screen, endemic mycoses serology, T-SPOT (Oxford Immunotech, Abingdon, United Kingdom), and the HIV antigen-antibody test [1].

On the 15th day of empiric antimicrobial treatment, the serum T-SPOT returned positive. Three days thereafter, the intraoperative AFB culture grew drug-susceptible *Mycobacterium tuberculosis*, supporting a definitive diagnosis of tuberculous spondylodiscitis or Pott disease. Vancomycin, cefepime, and metronidazole were discontinued, and daily ATT was started with rifampin 600 mg, isoniazid 300 mg, pyrazinamide 2000 mg, and ethambutol 1600 mg.

**Figure 1.**
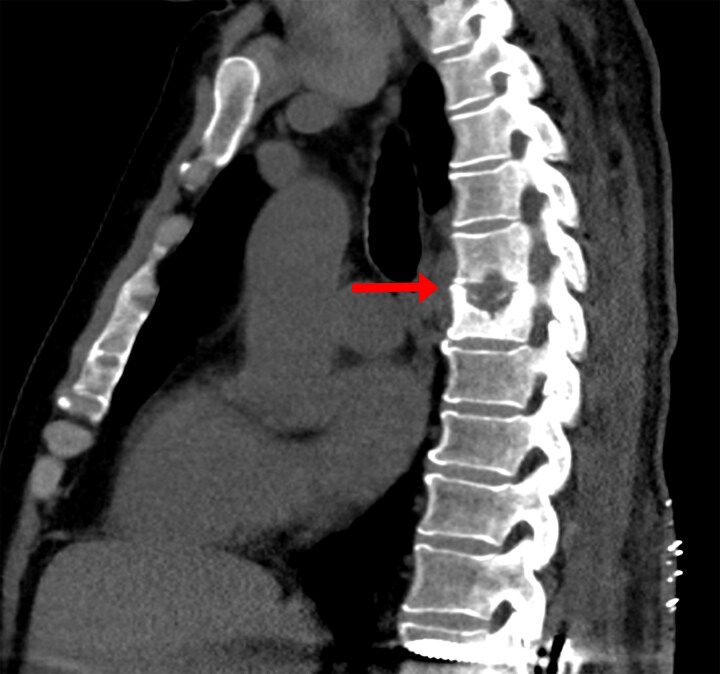
Noncontrast chest computed tomography in a sagittal view. The arrow highlights incidentally discovered lytic destruction of the T6 and T7 vertebral bodies.

**Figure 2.**
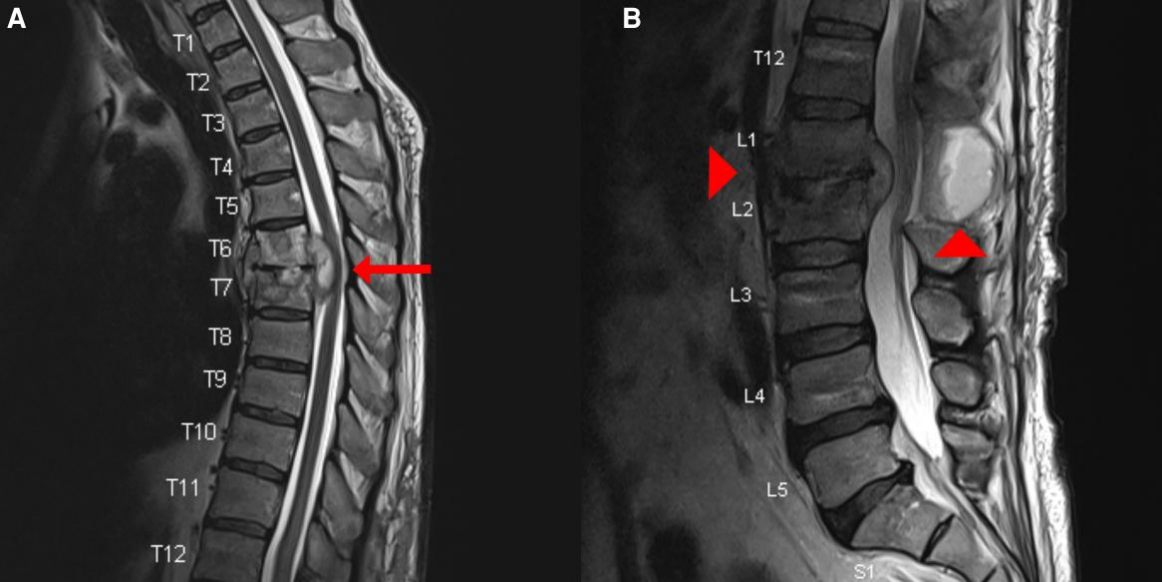
At the time of diagnosis, T2-weighted magnetic resonance imaging of the thoracic spine (*A*) and lumbar spine (*B*) in sagittal views. The arrow highlights spondylodiscitis of T6 and T7 with an associated epidural abscess causing mass effect on the spinal cord. The arrowheads highlight spondylodiscitis of L1 and L2 and a fluid collection at the laminectomy site.

**Figure 3.**
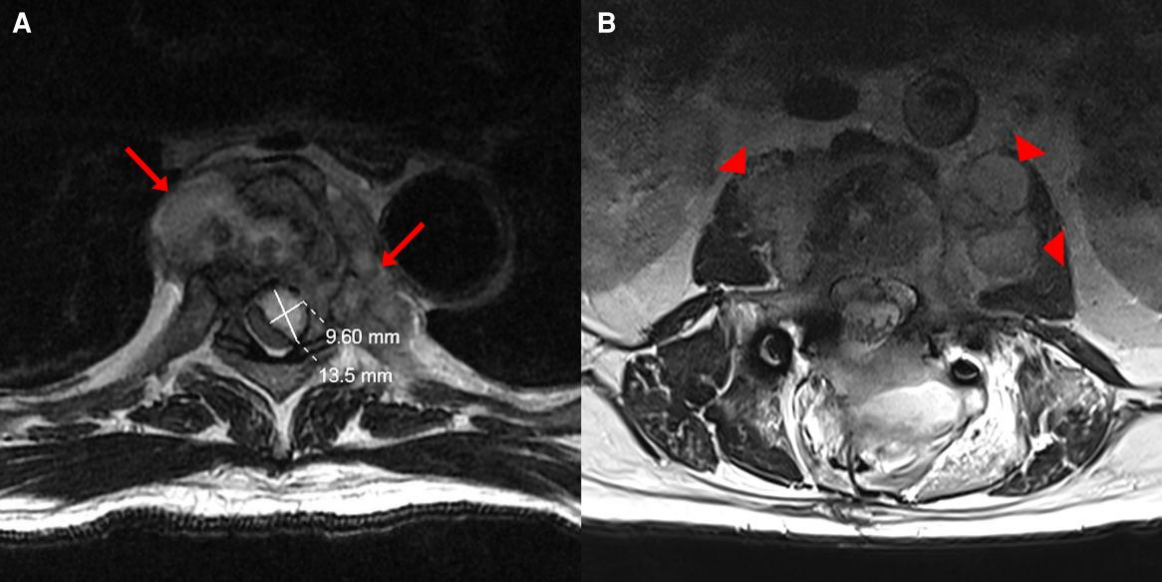
At the time of diagnosis, T2-weighted magnetic resonance imaging of vertebra T6 (*A*) and the evacuated vertebra L2 (*B*) in axial views. The arrows highlight paravertebral abscesses in the thorax. The cross mark outlines the dimensions of the thoracic epidural abscess as 9.60 × 13.5 mm. The arrowheads highlight the paravertebral abscesses at the psoas muscles.

A noncontrast chest computed tomography (CT) was done to assess for coexisting PTB. Although no pulmonary abnormality was noted, lytic destruction of the T6 and T7 vertebral bodies was incidentally discovered, raising concern for noncontiguous spinal disease (Figure 1). Therefore, a neuraxis MRI was done. The thoracic spine showed enhancement of the T6 and T7 vertebral bodies with associated paraspinal abscesses and an epidural abscess causing severe mass effect on the spinal cord. The lumbar spine showed persistent enhancement of the L1 and L2 vertebral bodies; prevertebral abscesses causing obliteration of the neural foramina from L2 to L4; and a fluid collection at the previously evacuated laminectomy site (Figures 2 and 3).

Neurosurgery debated decompression of the thoracic epidural abscess, given concerns about neurologic compromise. Since the patient had preserved motor strength on comprehensive neurological examination, they ultimately recommended nonoperative management with the use of ATT and a thoraco-lumbar-sacral-orthosis (TLSO). Additional neurological findings included decreased sensation at bilateral medial and lateral thighs, no myoclonus or saddle anesthesia, and a slowed gait requiring an assistive rolling walker. A lumbar puncture was deferred since the absence of fever, headache, or meningism lowered suspicion of TBM. However, given concerns about tuberculous involvement of the CNS, daily high-dose rifampin 900 mg and moxifloxacin 400 mg instead of ethambutol were started for improved penetration of the CNS [9, 10]. Daily prednisone 60 mg was also started to protect against TB-IRIS [4]. After 2 doses, daily prednisone was reduced to 40 mg due to the patient experiencing insomnia.

**Figure 4.**
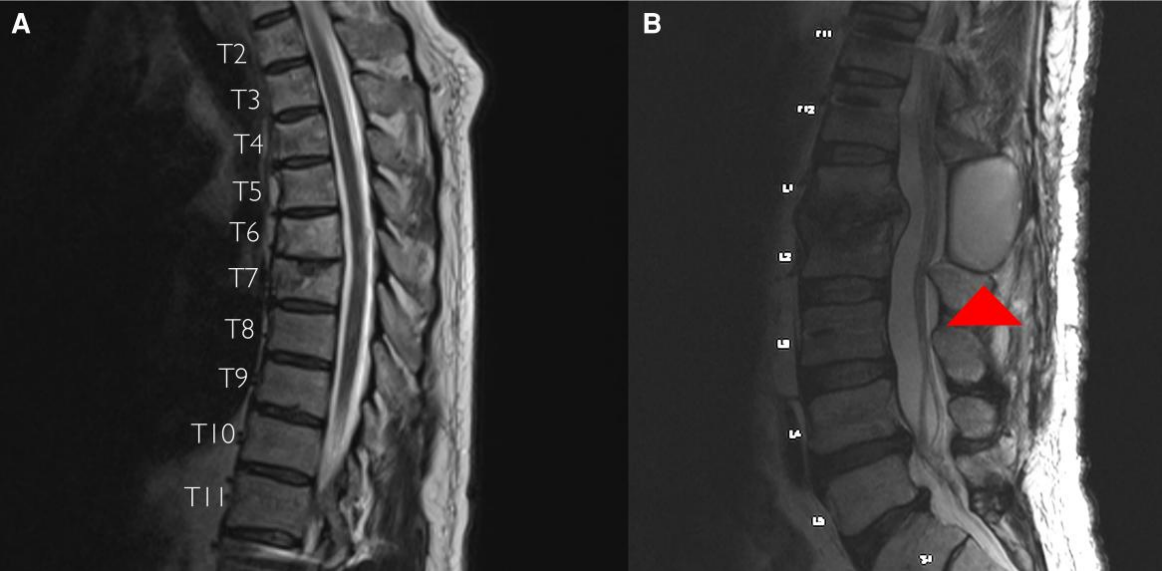
At month 9 of therapy, T2-weighted magnetic resonance imaging of the thoracic spine (*A*) and lumbar spine (*B*) in sagittal views. The arrowhead highlights evolving purulence at the lumbar laminectomy site, in the background of otherwise improving tuberculous infection.

Antituberculous medications were administered to our patient daily through directly observed therapy: high-dose rifampin, isoniazid, pyrazinamide, and moxifloxacin for 3 months, followed by high-dose rifampin, isoniazid, and moxifloxacin to complete 12 months. He reported gradual improvement in his lower extremity paresthesias. During serial neurological examinations, his gait progressed, but sensory impairments persisted at the thighs. Surveillance MRIs conducted at months 1, 3, 6, and 9 of therapy showed resolving spondylitis and abscesses, except for an evolving fluid collection at the lumbar laminectomy site, which approximately doubled in volume to a size of 60 mm × 47 mm × 36 mm (Figure 4). Ultrasound-guided aspiration was thus done at month 9, removing 30 mL of purulent fluid. Gram stain showed few WBCs with no organisms; AFB smear was negative; and the aerobic, fungal, and AFB cultures were sterile. The AFB culture was performed by inoculating the fluid specimen onto a Lowenstein-Jensen slant media and into a liquid-based Mycobacterial Growth Indicator Tube (BD BACTEC MGIT 960). Subsequent CT scans completed at months 11 and 18 showed interval improvement and appropriate positioning of his hardware. In addition, no complications of wound healing occurred at the low back. Neurosurgery therefore recommended removal of the TLSO brace.

Due to the patient's slowed clinical response and the potential for late-onset TB-IRIS in HIV-uninfected individuals, prednisone was tapered over 1 year [11, 12]. After 4 months, daily prednisone 40 mg was reduced by approximately 5 mg per month to a daily dose of 5 mg. To prevent adrenal insufficiency, endocrinology then switched prednisone to hydrocortisone 10 mg in the morning and 5 mg in the evening, which was successfully discontinued when the patient showed an appropriate response on cosyntropin stimulation testing. Daily famotidine 20 mg and thrice-weekly trimethoprim/sulfamethoxazole 800/160 mg were given to prevent gastric ulceration and opportunistic infection from *Pneumocystis jirovecii* while the patient was receiving 20 mg or greater of daily prednisone [13]. After the steroid taper, a dual-energy X-ray absorptiometry scan was done and showed osteopenia (T-score: –1.0). Endocrinology therefore recommended a single dose of intravenous zoledronate 5 mg. Surveillance imaging completed 18 months thereafter showed normal bone density per criteria of the World Health Organization [14].

On a final neurological examination, the patient had intact strength of the lower extremities, impaired sensation at bilateral medial and lateral thighs, no myoclonus, and coordinated gait without requiring an assistive device. Laboratory data showed an erythrocyte sedimentation rate of 16 mm/hour and a CRP of <0.5 mg/dL.

## DISCUSSION

Pott disease accounts for 1%–2% of all tuberculosis (TB)–related infections [15]. The annual incidence is 279 cases in the US [16]. Nonetheless, clinicians should consider Pott disease in patients with vertebral osteomyelitis and risk factors for TB, an initially nondiagnostic tissue biopsy, or clinicoradiographic features suggestive of tuberculous infection [1]. The thoracolumbar spine is frequently affected. Patients experience back pain (70%), fever (33%), weight loss (30%), and neurologic abnormality (19%) [17]. Kyphosis and paraplegia can occur in advanced cases [18]. MRI may show involvement of multiple vertebral bodies, thin-walled abscesses, and later-onset narrowing of the intervertebral disk [17]. Definitive diagnosis of Pott disease is challenging; growth of *M tuberculosis* in extrapulmonary tissue samples is paucibacillary and limits the yield of AFB culture (47%) and smear (25%–75%) [2, 7]. Moreover, non-tissue-based immune assays, including the T-SPOT and QuantiFERON-TB, cannot differentiate between latent or active tuberculous infection, and both have reduced sensitivity in various immunocompromised populations [19]. Authors have thus proposed an algorithm for presumed diagnosis based on clinical, serum, and radiographic findings [17]. Pyogenic spondylitis, brucellar spondylitis, fungal infections, and malignancies are other considerations on the differential. Unique aspects of our case included Pott disease in a US-born person and involvement of noncontinuous vertebrae, which is seen in up to 16% of cases [15].

TB-IRIS is an excess immune response to mycobacterial antigens released from the lysis of *M tuberculosis.* The phenomenon has been reported in 8%–43% of people with HIV (PWH) who begin antiretroviral therapy (ART) while receiving ATT, and in 2%–23% of persons without HIV who begin ATT [4, 6]. Among HIV-uninfected individuals, the prevalence of TB-IRIS is greater in cases of extrapulmonary TB than PTB, ranging from 16% to 50% [12]. Moreover, TB-IRIS has been observed later in persons without HIV [4]. In 1 study involving 122 cases of TB-IRIS in persons without HIV, median time to onset was 60 days after the start of ATT, with a range of 14–270 days [11]. On the other hand, among PWH who are receiving ATT, TB-IRIS generally occurs within 2–4 weeks of starting ART [20]. Although the mortality of TB-IRIS is low at approximately 3%, morbidity can be substantial [4]. Patients experience new, recurrent, or worsened TB-related signs, symptoms, and or radiographic findings [11]. In 2 single-center randomized trials involving PWH on ART and ATT, adjunctive prednisone given for 4 weeks reduced both the incidence and morbidity of TB-IRIS [5, 6]. However, there are no consensus guidelines on the prophylaxis or treatment of this immune phenomenon [4]. Furthermore, comparable trials are not available in HIV-uninfected individuals, and the use of steroids in this population to prevent or treat TB-IRIS requires case-by-case consideration [12].

Prednisone was started in our patient without HIV due to concern that paradoxical worsening of his abscesses may cause morbid neurological injury. Particularly, there were concerns of paradoxical reaction occurring at the thoracic epidural abscess, since it resided in an enclosed anatomic compartment and abutted the spinal cord prior to initiating ATT. This phenomenon did not occur; however, the expanding purulence at the lumbar laminectomy site while receiving treatment was a potential manifestation of TB-IRIS. Considering diagnostic criteria of TB-IRIS described by Geri and colleagues, our patient experienced radiographic worsening of tuberculous infection, despite initial improvements on ATT, and alternative explanations for this deterioration were not suspected [12]. Neither mycobacterial drug resistance nor nonadherence to ATT were noted, and cultures of the aspirated fluid specimen were sterile.

Prednisone was then tapered cautiously over 1 year because of the patient's slowed clinical improvement, the potential for late-onset TB-IRIS at other sites of infection, and as a potential therapy to mitigate morbidity associated with the patient's suspected paradoxical reaction. Prednisone was given additional consideration for use in our patient's case since tuberculous involvement of the CNS, particularly radiculomyelitis, could not be excluded with certainty. He reported lower extremity paresthesias; lumbar paravertebral abscesses abutted the neighboring neural foramina from L2 to L4; and associated radiculomyelitis has been noted in 44% of cases of tuberculous spondylitis [21]. Corticosteroids, administered between 4 and 8 weeks, improve survival in TBM and are thus believed to potentially benefit other tuberculous involvement of the CNS [3, 22]. Last, corticosteroids may be considered in cases of pericardial or ocular TB that have a high risk for inflammatory complications [23, 24].

The medical management of Pott disease derives from studies of PTB [2]. Health agencies recommend daily dosing of ATT for 6–9 months: an initial 2-month phase of rifampin, isoniazid, ethambutol, and pyrazinamide followed by 4–7 months of maintenance rifampin and isoniazid [23, 25–27]. Likewise, management of Pott disease with concomitant involvement of the CNS derives from studies of TBM [3]. In this scenario, agencies recommend extending the duration of treatment to 9 or 12 months [23, 25]. Our patient therefore received ATT for 12 months total, given concerns about potential tuberculous involvement of the CNS, including epidural abscesses encroaching upon the spinal cord and an inability to exclude radiculomyelitis. We departed from the guidelines, though, by starting high-dose rifampin and moxifloxacin instead of ethambutol since these medications better penetrate the CNS [9, 10]. Additional trials are still needed to assess whether this pharmacokinetic advantage improves outcomes in patients with TBM and or other tuberculous involvement of the CNS [9, 28]. In 1 single-center randomized trial involving 60 persons with TBM, an initial 2 weeks of high-dose intravenous rifampin (∼13 mg/kg) versus traditional oral rifampin (∼10 mg/kg) was associated with improved mortality at 6 months, whereas the use of moxifloxacin did not show a beneficial effect [29]. Conversely, a similarly designed trial involving 120 individuals with TBM noted superiority of levofloxacin versus rifampicin in reducing 6-month mortality [30]. Higher-powered contemporary and future clinical trials are investigating the effect of high-dose rifampin plus moxifloxacin or linezolid, another CNS-penetrant ATT, on outcomes in TBM [31]. Last, an antituberculous regimen containing intensified rifampin and a fluoroquinolone was given additional consideration for our patient since it may benefit individuals with isoniazid-monoresistant TBM [32]. Initially, susceptibility of the *M tuberculosis* isolate was unknown for our patient, and potential for isoniazid monoresistance was weighed. This tuberculous resistance pattern is commonest worldwide, and among individuals with TB in the United States, resistance to isoniazid has been noted in 8.4% of cases versus multidrug resistance in 1.4% [33, 34]. Despite this consideration of resistance, use of isoniazid in the antituberculous regimen was preferred given its potent, early bactericidal activity against susceptible *M tuberculosis* [23].

Due to the varying pharmacokinetics of antitubercular medications, health agencies and experts suggest that therapeutic drug monitoring may benefit certain persons with TB, including those with delayed response to treatment, severe illness, CNS involvement, drug resistance, drug–drug interactions, HIV coinfection, and malnutrition, among others [23, 35–37]. Last, on presentation to the outside facility, our patient underwent surgical correction of his spinal instability. Additional indications for surgery in Pott disease include progressive neurologic deficit, abscesses causing mass effect, and failed medical management [7]. Spinal instrumentation is considered safe during active infection given the paucity of biofilm formation by *M tuberculosis* [15, 38].

## Notes

***Author contributions.*** All authors contributed to the writing and editing of this work.

***Patient consent.*** Written informed consent was obtained from the patient.
